# Requirements for effective investigation and learning after suicide: the views of persons with lived experience and professionals

**DOI:** 10.3389/frhs.2025.1519124

**Published:** 2025-02-25

**Authors:** Elin Fröding, Charles Vincent, Boel Andersson-Gäre, Åsa Westrin, Axel Ros

**Affiliations:** ^1^ Jönköping Academy for Health and Welfare, Jönköping University and Region Jönköping County, Jönköping, Sweden; ^2^ Department of Experimental Psychology, University of Oxford, Oxford, United Kingdom; ^3^ Futurum, Jönköping Academy for Health and Welfare, Jönköping University and Region Jönköping County, Jönköping, Sweden; ^4^ Department of Clinical Sciences Lund, Psychiatry, Lund University, Lund, Sweden; ^5^ Office for Psychiatry and Habilitation, Psychiatry Research Skåne, Lund, Sweden

**Keywords:** suicide, suicide prevention, patient safety, investigation, improvement, mental health

## Abstract

**Objective:**

This study aims to provide a deeper understanding of what persons with lived experience and professionals with experience of patient safety, suicide research, and investigations consider to be most important in investigations of healthcare before suicide to learn and improve the care of suicidal patients.

**Method:**

This is a qualitative study based on 15 semistructured interviews with persons with lived experience of suicidality and professionals. Thematic analysis was used.

**Results:**

The persons with lived experience and the professionals agreed that a holistic approach to the investigations is crucial. They should embrace a longer period of time, involve family and significant others, integrate the perspective and expectations of the patient, and analyze factors of significance for suicidality, suicide prevention, and safety. There is a need to improve the investigations through the involvement of all stakeholders and actors, securing competence in the investigation team and prioritizing cases to investigate.

**Conclusions:**

Substantial changes in the approach and performance of investigations of suicide in healthcare are needed to make these investigations valuable for increasing the safety of the care of suicidal patients. A holistic perspective during the analysis is crucial for understanding the suicidal process, the interacting factors, and the care process preceding suicide. Competencies in suicidality, suicide prevention, and patient safety must be included in the analysis team to ensure high quality and relevance. To improve the value of these investigations, we suggest establishing a template based on current knowledge to ensure attention to variables of significance for a safe care of suicidal patients.

## Introduction

Suicide remains a global health problem; annually, approximately 700,000 people die by suicide (1). A large proportion of individuals taking their lives were shown to have been in contact with healthcare services close to their time of death, highlighting an area where work is needed within suicide prevention healthcare. There is a growing body of evidence for suicide prevention strategies in healthcare: identification and proper treatment of psychiatric disorders and abuse, psychotherapy, brief interventions, and safety planning (2, 3). However, despite efforts on suicide prevention, the decline in suicide rates has leveled off during recent decades in many countries (1), suggesting that new approaches and new interventions are needed. Learning from investigations of suicide cases could contribute to the development of such interventions.

Systematic reporting and investigations of incidents in healthcare to identify risks and improve patient safety have become widespread safety improvement strategies (4–6). The paradigm predominant across incident analysis in healthcare is a linear, cause-and-effect approach with a focus on deviations and nonadherence. However, the effectiveness of this standard approach has been questioned (7–9).

In Sweden, events of severe patient harm, as well as events involving the risk of severe patient harm, that could have been avoided if appropriate actions had been taken by healthcare professionals should be reported to the supervisory authority. The report must include an investigation of the case. The content of the investigation is regulated by law and requires an identification of the contributory causes of the incident and of service improvements that may prevent the reoccurrence of such an incident. Our previous review of investigations of suicide cases revealed that investigations often lack analyses of variables that are significant for suicidal behavior and suicide prevention (10). Furthermore, the same deficiencies and failures in healthcare have remained over the years, suggesting that current investigation strategies are insufficient to find interventions to support progress in suicide prevention in healthcare (10–12).

To become effective and valuable tools for improving healthcare, we suggested, in a previous paper, that the investigations must reflect the progress in the knowledge of suicide behavior, suicide prevention, and patient safety (13). Making efforts to understand the perspective of patients, family, and professionals experienced in suicide investigations should be an important step in progress and are in line with the recommendations of The National Confidential Inquiry into Suicide and Safety in Mental Health in the UK (14).

This study aims to provide a deeper understanding of what persons with lived experience of suicidality and professionals working with investigations of suicide consider to be most important to analyze in healthcare before suicide to learn and improve the care of suicidal patients and how this can be done. The core research questions were twofold: What is most important to analyze in investigations of healthcare before suicide, and how can this be done?

## Method

This paper reports on a qualitative interview study using a semistructured approach.

The authors are researchers and working professionals in psychiatry and suicidology (EF and ÅW), patient safety (AR, CV, and EF), and improvement in healthcare (BA-G).

### Participants

The characteristics of the responders are given in Table 1.

**Table 1:** Characteristics of the responders.

Characteristics	Number (*n*)
Women	*n* = 10
Men	*n* = 5
Lived experience of suicidality	*n* = 3
Family to deceased	*n* = 2
Researchers in suicidology	*n* = 4
Patient safety leaders	*n* = 3
Officials at supervisory authority	*n* = 3

#### Persons with lived experience of suicidality

Inclusion criteria: own experiences from surviving a suicide attempt or being a family member of a person deceased by suicide. The voluntary organization for suicide prevention “Suicide Zero” (http://www.suicidezero.se) in Sweden assisted in the recruitment of participants for this study. The head of the organization informed eligible members and sent contact information to those who showed interest in participating to EF. We made a strategic sampling, choosing five persons with different backgrounds and experiences, ages, and gender. EF contacted these persons in the way they wished and mailed written information about the study. Five participants were asked to participate and consented.

#### Professionals

Three groups of professionals were regarded as suitable responders: national leading suicide researchers, patient safety leaders, and officials at supervisory authorities (the National Board of Health and Welfare and the Health and Social Care Inspectorate). To ensure confidentiality, these three groups together are referred to as “professionals.” The inclusion criteria were extensive experience in suicide research or work with suicide investigations. The research team made a list of possible informants fulfilling the inclusion criteria from their professional networks from different geographical regions in Sweden. Invitation and further contact before the interviews were conducted via e-mail. Eleven possible participants were asked to participate, ten of whom accepted within a reasonable time and were thus included. Some of the included professionals were also working in the clinical frontline as psychiatrists, and some were working with investigations and analyses of suicides.

### Interviews

All participants provided voluntary informed consent to participate in the study. The participants chose the time for the meeting within a given time frame and received the prototype protocol to read before the meeting. The meetings were individual, except one with two officials from a supervisory authority in the same session. All the interviews were conducted by EF, using the video function on Zoom. The duration was 50–100 min, with an average of 72 min. All the participants agreed to be recorded to enable transcription of the interview. The interviews followed the interview guide (Table 2). The interview guide was developed by the research team and was structured around the core research questions. The interview guide was piloted with one patient safety leader before data collection (not included in the study) and was found to be feasible. The interviews were conducted in 2021. The interviews were transcribed verbatim by EF and the videos were thereafter deleted.

**Table 2:** Multicollinearity analysis.

Questions	
1	What is most important to analyse in the investigations of healthcare before suicide?
2	How can this be done?
3	How can we bring in the perspectives of the patient?
4	What time period do you think is relevant to analyse?
5	How to recognize warning signs for suicidality?
6	How to think about suicide risk assessments?
7	What are the key elements to analyse in the healthcare contacts?

### Data analysis

Systematic text condensation, as described by Malterud, was used in the analysis of the interviews, following four steps: (1) reading all the material to obtain an overall impression and identifying preliminary themes; (2) identifying units of meaning corresponding to aspects of participants' experience related to the method and contents in investigation of care at suicide, and coding for these units; (3) condensing and summarizing the contents of each of the coded groups; and (4) generalizing descriptions and concepts concerning the experiences of investigation of care at suicide, forming potential themes and subthemes (15).

The analyses were first performed by EF and AR together and then discussed and refined by the research team. Step 1 was performed by analyzing the data from the persons with lived experience and professionals separately. The preliminary themes turned out to be similar in the groups. Steps 2–4 in the analysis were then performed for the data as a whole, with an acute awareness of possible emerging differences between the groups. The text was read and reread several times to grasp the responders' experiences in relation to the study aim. A pragmatic semantic realist approach was used, assuming that what the interviewees said actually reflected their experiences. The potential themes were refined and defined until they were considered to reflect the coded data, coherence between the persons with own experience and the professionals, and the data set as a whole.

All the interviews were performed, transcribed, and analyzed in Swedish. The themes, subthemes, meaning units, and quotes were translated into English before the discussion with the research team.

## Ethics

This study was approved by the Swedish Ethical Review Authority in August 2021 (Dnr 2021-03701).

## Results

The main findings in the analysis were the same for the persons with lived experience and the professionals, but two more subthemes in the second theme were identified among the professionals (see below). We identified two themes, *a holistic approach* and *effectiveness of investigation*, covering five and four subthemes, respectively (Table 3). The themes and subthemes are interlinked and not mutually exclusive. In the text below, the quotes referring to persons with lived experience are labeled “LE,” and those of professionals with a “P.”

**Table 3:** Summary of themes and subthemes.

Holistic approach	Effectiveness of the investigation
Suicidality	The involvement of all current stakeholders
Time	The competencies of investigator leaders and analysis team
The system supporting the patient	Prioritized cases for extensive analysis (P)*
The patient's perspective and expectations	Guide a structured approach (P)*
The factors of significance in the care	

*P = this subtheme was identified among professionals only.

### Theme 1: holistic approach

The first theme, covering five subthemes, described the participants' perceptions of what the investigations of suicide cases should include in the analysis to be valuable for learning, understanding, and improving the healthcare of suicidal patients.

#### Suicidality

An analysis of the assessment of suicidality and suicide risk were regarded as central parts of the investigations by the participants, as the assessment could guide further decisions about care, treatment, and suicide-reducing interventions.

“We need to understand when suicidal thoughts arose and in what situations in life. Life events, external factors, what happened in connection with care but also, other factors in life, suicidality is linked to life events to the highest degree.” (LE3)

Several participants emphasized the need for the involvement of significant others (i.e., family, social services in the municipality, school staff, and employers) in suicide risk assessments. The suicidal person can be ambivalent in the acceptance of care and might underplay the risks.

“Here, the relatives have a role to play, the process outside the hospital, not just the care process. Maybe you can find warning signals here, the relatives may have different stories, and the patient can hide things from us.” (LE5)

In practice, this would imply a wider involvement of significant others in healthcare, which then must be accepted by patients and asked for by clinicians.

#### Time

Extension of the time period included in the analysis was described as one of the most important factors for enabling learning and understanding of the care process preceding suicide. The principal message from participants is that the investigation must extend back to the very signs of thoughts of suicide.

“What time period, that question is important, what is really wrong? It is important to know when it started. The time varies from person to person, of course.” (P2)

“I see the perspective from the start of a problem, whether it is financial problems, unemployment, sick leave, or whatever it is … in some cases a couple of months, sometimes several years. You should look from the point before you enter care, before the situation becomes acute.” (LE3)

“You need to look back on the whole care period. I think that illustrates what I name ‘chafes,’ that has been abraded over time, bit by bit. Something little wrong happens, not an enormous mistake, but a little wrong and then a little wrong again and then a break in the continuity. Small chafing which then in the end results in a rather hopeless situation.” (P1)

Analyses limited to including only the last contacts with healthcare could fail to uncover potential progressive degradations in care and in the patient's situation over time. Furthermore, an analysis of the process of care over time was pointed out as a source of learning both for what was effective and for what had failed.

“You can learn a lot from the care process. Learn what was effective and helpful, and when it failed. I think the time axis is extremely important, to avoid too much focus on the last months.” (P6)

The system supporting the patient, in this study understood as the healthcare units, other stakeholders such as employer and social services, and significant others that the patients face in their daily life and care, was emphasized by the participants to be analyzed as a whole. The participants described negative consequences for care from the strict boundaries between the stakeholders, with a focus on their own specific part only.

“I experienced that there was a gap between the stakeholders, primary care, hospital, and public services, such as bailiff authority. You did not see the whole picture. You are so inside your own little box, I think you could gain a lot from that, to really involve all the different stakeholders in the investigation, not only the emergency care or the final stage. What I am thinking of, it is not only the care contacts, but other contacts that could also make a difference … other actors who could catch up, or hear. It may be easier to talk about some things at the job centre or at the debt counseling services.” (LE3)

The investigations should analyze the system of the patient as a whole, including the cooperation between the stakeholders, to gain an understanding of the interplay and identify possibilities for improvement.

The patient's perspective and expectations of how the patient experienced the provided healthcare, confidence in care, and how the care managed to meet the patients' expectations and needs were considered significant aspects of the analysis to understand the individual process and suicidal behavior.

“In what way has the patient been allowed to express what he wants? How does the patient perceive the treatment, and the therapist? Evaluation together with the patient is an important part of the care.” (P6)

The ability of professionals to listen to and meet the patients' needs was seen as crucial for maintaining patient adherence to the provided care.

“We ignored attending some appointments, even me! Because the care did not respond to our needs. However, it does not say so in the medical records; it is documented in other terms … I told them that we will not attend the meeting if they do not have a plan for the visit. Missed appointments are more important than you can imagine.” (LE1)

Missing appointments should be considered signs of disconnection and insufficient trust in healthcare, highlighting the need for care to have a plan for how to act when this happens.

The factors of significance in the care provided involved assessing both the strengths and the weaknesses of the care provided, including the skills and abilities of the staff involved.

Sufficient knowledge and training in professional tasks are critical for the clinicians' ability to achieve and maintain competencies in professional performance. An analysis of the competencies of staff involved in the care process was suggested for inclusion in the investigations.

“The competence issue among the staff is an important part of the investigation.” (P4)

The participants found that attention should be given to parts of care that were successful as well as to failures.

“Both what has worked well and badly should be highlighted; it is absolutely necessary to put it into words; to create a balance, it will be a patient safety risk in itself if you change the care after an event that rarely occurs.” (P5)

“Healthcare focuses too much on what is bad; you put a lot of effort into it, but the periods when it actually is stable should also be taken into consideration.” (LE4)

An analysis of stable periods could reveal useful coping strategies, and the need to learn from those strategies was highlighted by both the persons with lived experience and the professionals. Furthermore, the conclusions and findings should be valued in relation to the risks.

### Theme 2: effectiveness of the investigation

The second theme, *effectiveness of the investigation*, covers four subthemes and describes how the investigation should be performed to fully understand and explore the identified areas in the first theme.

The involvement of all stakeholders of relevance to the patient in the investigation process was highlighted to be crucial for managing a holistic approach, representing a different way of performing investigations compared with the standard approach of today. Individuals who die by suicide often suffer from multiple diseases and different problems, such as socioeconomic problems, resulting in contact with multiple professionals, healthcare providers, and social services. To understand the whole process of given care, all involved stakeholders need to be included in the investigation:

“We need to make wider analyses and involve all stakeholders.” (P10)

“I think you should do much more thorough investigations … interview other actors, involve school, private actors … the investigations should be performed more thoroughly by an external investigator, include several actors and embrace a longer time period.” (LE1)

In addition to staff, family and significant others of the patient were suggested to be possible informants in the analysis to capture the whole picture in the investigation.

“Families should always be included in the investigation, interviewed, and not just submit comments. It is resource-intensive to involve everyone around the patient, but that is what is required if we are to move any forward with the investigations.” (P6)

In practice, the family and other stakeholders should be actively involved in the analysis process and not restricted to readers of the finished investigation.

The competencies of investigator leaders and the analysis team in suicidology and healthcare were highlighted as prerequisites for making the investigations valuable and ensuring sufficient quality of the investigations. Furthermore, the investigator leaders should be external leaders and independent of the involved units; local investigators might face difficulties in being objective and endeavoring to preserve interpersonal relationships.

“The analysis leader should be someone who really knows the areas of suicide and risk assessment, and who dares to get into the task, an external investigator.” (P6)

“Of course, substantial medical competence and nursing competence are needed for these investigations. Real competence is required.” (LE5)

To enable an analysis of the often multifaceted complexity of problems of suicidality, investigations need to involve team members with different competences and perspectives.

“It is important with multidisciplinary teams to get forward, we have different views, and we see different things.” (P9)

In practice, this highlights the need for healthcare to invest in education and training of investigators and to involve experts in analysis teams to manage high-quality investigations of complex incidents.

#### Prioritized cases for extensive analysis

This subtheme was found among the professionals. The complex cases with several involved caregivers were considered to have the greatest potential for learning and were suggested to be prioritized for extensive analysis.

“We can probably learn most from the complex cases, with several caregivers involved, they involve so many areas, and how do we interact, what structures do we have where we can meet?” (P2)

However, the consequences of such triage were also problematized. Analyses of all suicides enable an understanding of changes in the system over time.

“You have to understand the overall picture too, you cannot leave any suicides behind. Of course you need to deepen the analysis when there are obvious shortcomings, but it is difficult to deselect cases, you can lose something in the whole as well, you need to follow the development over time too.” (P6)

For this purpose, less extensive investigations could be considered in less complex cases.

The investigation process was also suggested to be a useful tool after suicide attempts to make a ground for the content in a crisis plan.

“I think it would contribute after suicide attempts as well, to use the investigation actively in healthcare, in the development of a crisis plan.” (P10)

In this way, a crisis plan could enable engagement from the broad network of significant actors around the person and help them understand their role and integrate learning from prior crises and incidents into the plan.

#### Guide a structured approach

This subtheme was found among the professionals. The use of a template that stipulates what the investigations should include could ensure attention to variables of significance and serve as a guide for the investigators.

“The investigators must be helped to focus on relevant issues in the investigations.” (P3)

“It would be an advantage to have a template that guides you a bit.” (P4)

The template was suggested to include checklists and issues of significant importance to consider in the analysis of a suicide incident as patient harm.

## Discussion

A holistic approach to the analysis of suicide was considered to be crucial for understanding the process preceding suicide. This understanding was highlighted to identify factors of real importance for safe care for suicidal patients and was essential for making the analyses valuable for progressing suicide prevention in healthcare.

Substantial changes in the approach and performance of investigations of suicide are also needed to meet the criteria we found in this study. On the basis of the findings of the analyses, we suggest nine actions to increase the quality of investigations that should be implemented in clinical practice, as presented in Table 4. These actions should be seen as challenges to and adaptations in standard approaches in suicide investigations.

**Table 4:** Actions making the investigations of suicides related to healthcare more valuable.

Action	Motivation
(1) Analyze the management of suicide assessments.	The assessments often serve as guides for decisions of healthcare and suicide-reducing interventions.
(2) Extend the analysis to the very time when the disease or relevant problem first started.	Crucial to understand the suicidal process, the interacting factors, and the care process preceding suicide.
(3) Analyze the system of the patient as a whole.	Enables understanding of the interplay between patient, next of kin, and stakeholders to identify possibilities for improvement in cooperation.
(4) Make efforts to understand how the patient experienced healthcare and how care managed to meet the needs of the patient.	Trust in healthcare and trustful communication are essential for adherence and influences the outcome of healthcare.
(5) Focus the analyses on the areas of importance for safe care and key events in the suicidal process.	Makes the investigation legible and eases identification of areas of improvement in suicide prevention.
(6) Integrate the experiences of family and significant others in the analyses.	Contributes to understanding the reality that the patient faced and managed in daily life and facilitates learning from how the care managed to meet the expectations and needs.
(7) Include expertise and broad experience in the analysis team.	Ensure good quality and relevance of the investigation.
(8) Save extensive analyses for the most complex suicide cases.	Cases with several involved caregivers were considered to have the largest potential for learning.
(9) A structured approach should guide the investigations.	A template based on all other actions should improve the investigations.

We realize that the holistic approach to investigation with the involvement of family and other stakeholders proposed here is time-consuming. We suggest that cases with the potential for new learning and revealing problems not highlighted in the current system should be prioritized for this extensive analysis and that fewer resources be spent on less complex cases, in line with the previous work of Vincent and Amalberti (16, 17). Furthermore, we suggest that the team takes advantage of the known outcome. The analysis should focus on the suicidal process, areas significant for safe care, and what learning and conclusions can be drawn on broad lines (12) and actively avoid being caught in irrelevant details littering the investigations with details that may obstruct seeing the whole picture. A holistic approach has also been suggested to benefit the assessment and management of patients at risk of suicide by shifting from prediction to therapeutic suicide risk assessment (18–20).

Integration of the experiences of the families and significant others in the analyses should become the default option, with the important provision of considering psychological and emotional timeliness. Families have been shown to contribute to a better understanding of work, as done in clinical practice, and strengthen the learning potential in resilience (21). A review concluded that most patients and families value being involved in incident investigations, but it is important to be flexible and sensitive to both clinical and emotional aspects of the investigation to avoid compounding harm (22). Studies have shown that performing interactive investigations with families can meet resistance from professionals (23, 24). One reason could be the shame and self-blame that healthcare professionals share with family post suicide, illustrating ‘second victimhood' (25). This illustrates that involvement requires careful professional respect for the involved staff, the family, and the deceased and awareness of the psychological impact that the analysis can have on all involved individuals (26). Applying a restorative just culture with support and protection against blame and inappropriate guilt at suicide has been shown to ease participation, learning, and progress the work with suicide prevention in healthcare (27, 28). We recommend that family and healthcare professionals directly involved in the care of the deceased contribute their perspective and reflections to the analysis but not be members of the analysis team.

In times when healthcare providers are struggling with staffing, recruiting, and keeping experienced personnel, it might not seem obvious that these persons should spend time investigating adverse events instead of meeting patients in need. However, adequate competencies and experience in the investigation team are critical for the analysis to become valuable (8, 27). High-quality investigations have the possibility of gaining knowledge that can be implemented to increase the safety of care for future patients. The analysis of care and mapping of social networks are suggested to be valuable after suicide attempts and to strengthen healthcare. This possibility could be developed further in a separate future study.

The findings of this study provide a deeper understanding of the details of the analysis performance in suicide cases and are in line with the changes in the performance of investigations reported in our previous literature review (13). We suggest the establishment of a template based on current knowledge and research on suicidology and patient safety that stipulates what the investigations should analyze to ensure attention to variables of significance for the safe care of suicidal patients. A template could serve as a guide for the investigators and enable control of healthcare risk management over time, with the possibility of making the analyses more resource-effective.

### Strengths and limitations

There are several strengths and limitations of this study that should be taken into consideration in the interpretation of the results. The preunderstanding of the first author as a consultant psychiatrist working with suicidal patients for two decades provided great advantages in the understanding of the study results, but also increased the risk for confirmation bias.

The depth of experience of various types of participants is a great strength, but the small sample size is a limitation, and we realize that other opinions and perspectives, which may not be recognized in this study, are possible. However, the limited sample size enabled us to maintain a persisting overview of the whole data set along with the individual interviews during all steps of the analysis, and we regard the number and distribution of responders to be adequate to fulfil the purpose of this study. We made efforts to include men and women of different ages and from different parts of the country. All the responders were Swedish, and the context of their experiences was within Sweden, which must be considered with respect to transferability. Furthermore, all were strikingly engaged in the discussed issues and urgent to share their experiences in a constructive way. In future research, we suggest that efforts should be made to ensure diversity, involving people not that strongly committed and who might have different perspectives. Even though this study focused on investigations after suicide, we believe that our findings might be transferable to investigations into other types of patient harm, which could also be tested, evaluated, and adapted in practice in future research coproduced with identified stakeholders.

## Conclusions

Substantial changes in the approach and performance of investigations of suicide in healthcare are needed to make these investigations valuable for increasing the safety of the care of suicidal patients.

A holistic perspective in the analysis is crucial for understanding the suicidal process, the interacting factors, and the care process preceding suicide. Competencies in suicidality, suicide prevention, and patient safety must be included in the analysis team to ensure high quality and relevance.

To improve the value of investigations, we suggest establishing a template on the basis of current knowledge to ensure attention to variables of significance for the safe care of suicidal patients.

## Data Availability

The original contributions presented in the study are included in the article/Supplementary Material; further inquiries can be directed to the corresponding author.
